# Oncocytic Papilloma

**DOI:** 10.1016/S1808-8694(15)30797-7

**Published:** 2015-10-19

**Authors:** Michel Cyrino Saliba, Vinícius Antunes Freitas, Eduardo César Dolabela de Moraes, Fabrícia Leandro de Barros, Roberto Eustáquio Santos Guimarães

**Affiliations:** 1 General practitioner, otorhinolaryngology medical resident, Núcleo de Otorrino, BH; 2 General practitioner, otorhinolaryngology medical resident, Núcleo de Otorrino BH; 3 General practitioner, otorhinolaryngology medical resident, Núcleo de Otorrino BH; 4 General practitioner, otorhinolaryngology medical resident, Núcleo de Otorrino BH; 5 Certified (livre-docente) professor, USP - Ribeirão Preto. Adjunct professor, Faculdade de Medicina da UFMG. Núcleo de Otorrino, BH

**Keywords:** oncocytic papilloma, schneiderian papillpma, nasal and paranasal tumors

## INTRODUCTION

The oncocytic papilloma, also named cylindrical cell papilloma, is a rare neoplasm of the nose and paranasal sinuses. It is derived from the Schneiderian membrane, an ectodermal mucosa that lines the nose and paranasal sinuses.^1^, ^2^, ^3^, ^4^, ^5^

Three types of papillomas develop from this membrane: the inverted, fungi-form and oncocytic types.^1^, ^2^, ^3^^,^^5^ The first two are responsible for about 45 to 50% of cases each;^1^ oncocytic papillomas are diagnosed in 3% to 5% of cases.^1^, ^2^, ^3^, ^4^, ^5^ These three varieties are known as Schneiderian papillomas.

The oncocytic papilloma is associated with the squamous cell carcinoma in about 15% of cases.^1^, ^2^, ^3^^,^^5^

## CASE REPORT

A retired female patient aged 60 years presented at our clinic having been referred for postoperative follow-up. She had sought an otorhinolaryngologist initially because of a complaint of post-nasal secretion that had lasted many years. The patient was atopic, with intermittent bronchial asthma and allergic rhinitis. She did not smoke or consume alcohol.

**Figura 1:**
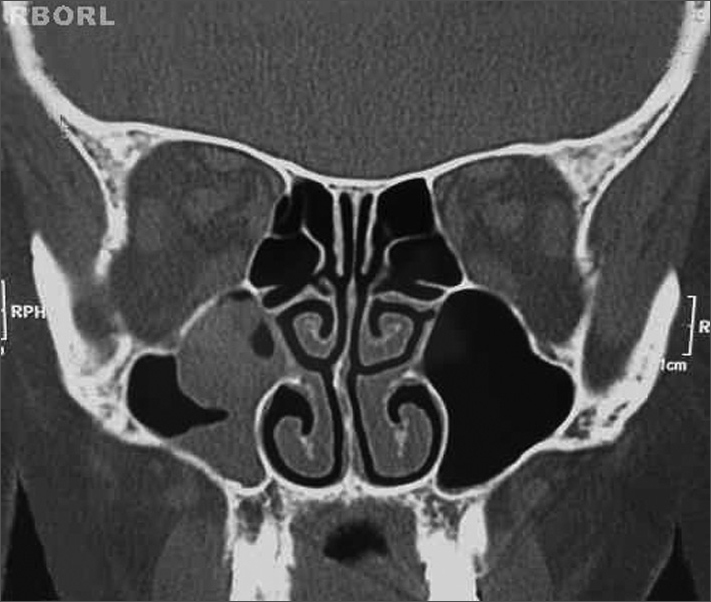
Oncocytic papilloma. Coronal CT showing an opacified right maxillary sinus.

Fibronasolaryngoscopy revealed that the nasal fossae and respective meatuses were patent. Computed tomography of the sinuses showed that the mucoperiostal lining of the right maxillary antrum was thickened; also present was a rounded dense image that did not involve other sinuses or bone. The patient had been referred to surgery, which she chose not to undertake at the time. About three months later she returned to continue therapy. A second computed tomography of the sinuses showed that the mucoperiostal lining of the wall of the right maxillary sinus was irregularly thickened; there was a rounded polyp-like formation inside the maxillary sinus, which extended to the ipsilateral infundibulum, which was partially obliterated. The other facial sinuses were unaltered (Fig. 1).

The patient underwent surgery: the Caldwell Luc sinusectomy, with removal of the entire mucosa of the right maxillary sinus (safety margin); there was abundant mucin and a dark secretion suggesting fungal sinusitis. Frozen biopsy was not done, since this procedure is not available at the clinic.

The material removed at surgery was sent to the pathology department. Macroscopic examination showed light brown polypoid tissue fragment. The microscopic examination revealed a nasal papillomatous lesion consisting of papillae and oncocytic cylindrical cell with occasional microcysts containing mucus or neutrophils, and sinus mucosa with a moderate chronic lymphocytic infiltrate. No signs of malignancy were found. The diagnosis was Schneiderian papilloma, oncocytic type, and non-specific chronic sinusitis.

The patient is being followed up to control recurrences.

## DISCUSSION

The clinical presentation of this disease is not well defined because few cases have been published in the literature. About 20 to 35 cases have been reported to date (the actual number varies depending on the report).^5^ Generally, these patients are in the fifth decade of life;^1^^,^^2^^,^^4^^,^^5^ there is no sex or race predominance.^1^^,^^2^^,^^4^^,^^5^ Unilateral nasal obstruction is the most common symptom.^1^, ^2^, ^3^, ^4^ Other symptoms that have been described are unilateral epistaxis^1^, ^2^, ^3^, ^4^, ^5^ and pain.^1^^,^^4^^,^^5^ The duration of these symptoms ranges from months to years, depending on the report.^1^, ^2^, ^3^, ^4^, ^5^ The epithelium may undergo malignant transformation, resulting in different types of invasive carcinomas. All reports have shown that lesions originated from the lateral wall of the nose and the maxillary or ethmoidal sinuses.

Imaging (radiographs or computed tomography of the facial sinuses) shows changes within the ipsilateral nasal sinus. The most common findings are an opacification associated with low-density intranasal tissue.^4^^,^^5^ Bone destruction suggests concomitant malignancy.^1^^,^^4^^,^^5^

The treatment is surgical always.

Postoperative chemotherapy or radiotherapy may be necessary, usually when there are signs of associated malignancies.^1^^,^^3^^,^^4^

Postoperative recurrence is estimated at 25 to 35%, usually on the surgical site of the previous disease.^1^^,^^3^^,^^4^^,^^5^

## COMMENTS

The patient sought our clinic for post-surgical follow-up. She had undergone conservative surgery, in which not all of the mucosa was removed (as recommended), which leaves the door open for recurrences.

We plan rigorous follow-up, since there is a high probability of recurrence. Furthermore, there is a 15% association with squamous cell carcinoma.
